# Experiments on the Recurrent Sensibility of the Anterior Roots of the Spinal Nerves

**Published:** 1861-04

**Authors:** Austin Flint

**Affiliations:** Professor of Physiology and Microscopy in the New Orleans School of Medicine


### SELECTIONS.

*Experiments on the Recurrent Sensibility of the Anterior Roots of the Spinal Nerves.* By AUSTIN FLINT, Jr., M. D., Professor of Physiology and Microscopy in the New Orleans School of Medicine.

There are few facts in physiology better known and more generally admitted, than those which have referrence to the functions of the two roots of the spinal nerves. The anterior roots are motor, and the posterior roots are sensitive. Like most important discoveries, however, the credit of its authorship has been somewhat disputed, though it is now usually conceded to Sir Charles Bell. It is a curious fact, neverthelesss that in 1809, two years before the appearance of Sir Charle, Bell's first essay, Mr. Alexander Walker, an English physician, advanced the idea that the two roots of origin of the spinal nerves had different functions; but he supposed that the posterior roots were motor, and the anterior roots senitive. To support this view, which was a mere supposition, Walker brought neither physiological nor pathological proofs; but in 1811, Charles Bell made the great discovery with which his name will be forever connected, *i. e.*, that the posterior roots conducted sensations, and that their irritation produced no movements, while the anterior roots were motor, as proved by the occurrence of convulsions when they were stimulated. Bell, however, was not a vivisector: his experiments were chiefly on rabbits, which he suddenly killed, opened the spinal canal, and irritated the posterior and anterior roots of the nerves—the experiments resulting, as just stated, in convulsion of muscles when the anterior roots were stimulated and none when the same irritation was applied to the posterior roots. These experiments made a great sensation in the physiological world, and were repeated by eminent French and German physiologists, among whom were Müller, Valentin, Magendie and Longet. Longet, especially carried Bell's experiments on the nerve-roots to the columns of the cord, and demonstrated that the anterior columns were motor, while the posterior were sensitive. He also made a number of confirmatory experiments upon the roots of the nerves in *living* animals.

But the experiments which are chiefly to be noticed in the present communication were made by Magendie; who, though many physiological facts had of course already been arrived at by experiment, may be said to be the father of the experimental school of physiology. He made experiments, which were published in 1822, upon the roots of the spinal nerves, and found that, while the posterior roots were purely sensitive, the anterior roots were not, in all of his observations, purely motor, but sometimes possessed a slight degree of sensibility. These experiments he repeated in 1839, and was able to establish at that time a certain degree of sensibility in the anterior roots of the spinal nerves; and also the fact that the facial, possessed some sensibility, and that this was derived from the fifth pair. This he called the "recurrent sensibility;" and these experiments tended to show that the views of Sir Charles Bell and his followers had been too exclusive—that the anterior or motor roots possessed a certain degree of sensibility, though not as such as the posterior roots. Here we meet with a very curious event in the history of the recurrent sensibility: Magendie's experiments, being in a manner opposed to the views of Bell, which were then universally received, attracted considerable notice; but when, after 1839, he attempted to repeat them, he utterly failed, and finally abandoned the ground that he had taken—one of the numerous examples of the honesty of description which mark the experiments of this distinguished physiologist. It was not until 1846 that Bernard, who, in experimentation, seem to divine intuitively all sources of error, revived the recurrent sensibility, and succeeded in again demonstrating it. He remembered that in 1839, the experiments for the lectures of Magendie were prepared in the morning, and that the animals used for the purpose of demonstrating the recurrent sensibility were thus permitted a period of repose before demonstrations were made during the lecture, which took place in the afternoon. He himself had made operations on the spinal cord and roots of the nerves, and had found that after the operation of opening the spinal canal, which is exceedingly painful and tedious, the general sensibility of the animal was blunted. The state of the nerves, then, was more natural after the animal had been permitted to recover from the effects of the operation, than immediately after the exposure of the roots. Carrying this idea into practice, he opened the spinal canal in dogs and allowed them two or three hours' repose before he made his observations on the roots of the nerves. In experiments conducted in this way, especially in dogs that were vigorous and well nourished, he always found the anterior roots of the nerves sensitive. He found, also, that the sensibility was derived from the posterior roots, for the division of these immediately abolished the sensibility of the anterior roots. He made, in addition, some interesting observations upon the disappearance of sensibility from exhaustion, anæsthetics, or other causes. He showed that sensibility first disappeared in the anterior roots, then in the periphery, and last in the posterior roots. When the sensibility reappeared, it was first manifest in the posterior roots, then in the periphery, and last in the anterior roots. These experiments of Bernard have fully established the facts of recurrent sensibilty; but, as far as I am aware, have only been repeated and confirmed by M. Schiff, an eminent Parisian physiologist.

As I have lately had occasion to repeat these experiments at the New Orleans School of Medicine, and demonstrate to the class, in my regular course of lectures at that institution, the recurrent sensibility, I have thought the subject sufficiently important and interesting to put upon record my own experiments confirmatory of the views held by Magendie in 1839, afterwards abandoned by him, but confirmed in 1846 by Bernard.

Bernard's experiments were made before the use of ether as an anæsthetic; but Schiff, who repeated these experiments at a later date, always made use of ether in the operation of opening the spinal canal. This of course abolishes pain dur- the preliminary operation; and, in the course of one or two hours, the animal entirely recovers from it effects and is ready for the observations on the nervous roots. It is best to select a vigorous, healthy dog for the operation, as the sensibility is then much more marked. The most convenient situation, also, at which to open the spinal canal is in the lumbar region at the point of junction of the iliac bones with the spinal column.

*Experiment I.*—Feb. 15, 11, A. M. A vigorous, medium-sized dog was completely etherized, placed on his belly on the table, with a billet of wood under the lumbar region to make this part of the spinal column prominent. The hair was then cut from the parts to be incised, and a longitudinal incision, about four inches in length was made, just to the left of the spinous processes of the lower lumbar vertebræ. This incision was then carried down by the sides of the spinous processes to their junction with the laminæ. The laminæ of the fifth, sixth and seventh lumbar vertebræ were then denuded of their muscles and were cut through vertically by a fine saw carried as near the spinous processes as possible. The laminæ were then divided near the transverse processes by a cut of the saw parallel to the first, but directed obliquely inwards. (There is danger in making this second cut, of wounding the nerves as they emerge from the spinal canal, and also of opening the vertebral sinus; this may be avoided by cutting with great care and not going through the laminæ entirely, but breaking them off by prying with a chisel introduced into the cut next the spinous processes.) In opening the spinal canal the roots of the fifth pair of lumbar nerves were divided. The roots of the sixth pair, however, were intact. These roots were separated carefully by a delicate steal blunt-hook, threads were passed beneath them, the wound was closed by sutures, and the animal set at liberty. After recovering from the effects of the ether, it was discovered that his left posterior extremity was partially, though not completely paralyzed, owing to the injury to the nerves during the operation of opening the spinal canal.

Feb., 16, 11, A. M. Twenty-four hours after the operation, the wound was opened; the discharges, which had been considerable, were removed, and irritation, by means of pinching with forceps, was applied to the roots of the nerves.—*Both roots were sensitive*, as manifested by the cries of the animal, but the sensibility of the posterior root was by far the more acute.

The wound was then again closed and the same experiments were repeated before the medical class, at 2, P. M. The sensibility of the posterior root was then acute, but that of the anterior root had considerably diminished, probably on account of exhaustion produced by the previous experiment.

*Experiment II.*—Feb. 16, 11½, A. M. The dog used in this experiment was a larger, younger, and more vigorous, dog than the one used in Experiment I. The animal was etherized, and the incisions made denuding the spinous processes and laminæ of the sixth and seventh lumbar vertebræ, as in Experiment I, with the difference that the operation was made on the right side. The laminæ of the sixth and seventh lumbar vertebræ were then removed, without wounding the roots of any of the nerves. This was done by making the cut with the saw farthest from the spinous processes extend only partially through the bone, and then removing the laminæ by prying them off with a chisel. The roots of the sixth lumbar nerves were then isolated, and threads of fine silk were passed beneath them. The wound was then closed, and the animal set at liberty. The operation lasted about three quarters of an hour.

2, P. M. The animal was exhibited to the medical class, and the following observations made before them. The slightest touch of the posterior root produced intense pain, as manifested by the cries of the animal. Upon pinching the anterior root, the animal cried, evidently suffering pain, though not so intensely as when the posterior root was barely touched. Care was taken in irritating the anterior, not to touch the posterior root. The posterior root was then divided, its section causing intense pain, and the anterior root was again irritated. Now, however, *its sensibility had entirely disappeared*, and it could be contused in the roughest manner without producing any evidence of suffering.

The operation for the purpose of exposing the spinal cord is quite difficult and tedious, on account of hæmorrhage, which is sometimes abundant, and the difficulty to avoid wounding the roots of the nerves and opening the vertebral sinus. These can be avoided, however, with a little practice. The instruments necessary for opening the spinal canal are a small saw, a Hey's saw, a pair of small bone-nippers, and a chisel. When the laminæ of the vertebræ have been removed, the spinal cord is exposed, surrounded by a certain quantity of fat, which should be carefully removed with the forceps. A pair of small, blunt, steel hooks are then necessary, for the purpose of isolating and catching up the roots of the nerves. It is best, then, to pass a fine thread under them before closing the wound, so as to be able easily to find them again. The wound is then to be closed, and the animal allowed to repose for two or three hours, when it will have recovered entirely from the effects of the operation, and the roots of the nerves will have regained their normal sensibility.

I have thus detailed two experiments, which show, in a marked manner, especially Experiment II, that the anterior roots of the spinal nerves are not exclusively motor, but that they possess a certain degree of sensibility; that this sensibility is recurrent, or is derived from the posterior or sensitive roots, because, after the division of these roots, it is immediately lost. In this I have confirmed the experiments of Magendie, in 1822 and 1839, experiments which he failed to repeat with success since that date, which were repeated in 1846 by Bernard, and later still by Schiff; but have never been repeated, as far as I am aware, in England or this country. In these experiments I have attempted to show nothing beyond the recurrent sensibility, and its derivation from the posterior or sensitive roots.—*N. O. Medical Times.*

